# CRISPR/CAS9-mediated knockout of Abi1 inhibits p185^Bcr-Abl^-induced leukemogenesis and signal transduction to ERK and PI3K/Akt pathways

**DOI:** 10.1186/s13045-020-00867-5

**Published:** 2020-04-10

**Authors:** James Faulkner, Peixin Jiang, Delaney Farris, Ryan Walker, Zonghan Dai

**Affiliations:** Department of Internal Medicine, Texas Tech University Health Sciences Center School of Medicine, 1406 Coulter St, Amarillo, TX 79106 USA

**Keywords:** Abi1, Bcr-Abl-positive B-ALL, Leukemogenesis, Actin cytoskeleton, Drug resistance

## Abstract

**Background:**

Abl interactor 1 (Abi1) is a downstream target of Abl tyrosine kinases and a component of the WAVE regulatory complex (WRC) that plays an important role in regulating actin cytoskeleton remodeling and membrane receptor signaling. While studies using short hairpin RNA (shRNA) have suggested that Abi1 plays a critical role in Bcr-Abl-induced leukemogenesis, the mechanism involved is not clear.

**Methods:**

In this study, we knocked out Abi1 expression in p185^Bcr-Abl^-transformed hematopoietic cells using CRISPR/Cas9-mediated gene editing technology. The effects of Abi1 deficiency on actin cytoskeleton remodeling, the Bcr-Abl signaling, IL-3 independent growth, and SDF-induced chemotaxis in these cells were examined by various in vitro assays. The leukemogenic activity of these cells was evaluated by a syngeneic mouse transplantation model.

**Results:**

We show here that Abi1 deficiency reduced the IL3-independent growth and SDF-1α-mediated chemotaxis in p185^Bcr-Abl^-transformed hematopoietic cells and inhibited Bcr-Abl-induced abnormal actin remodeling. Depletion of Abi1 also impaired the Bcr-Abl signaling to the ERK and PI3 kinase/Akt pathways. Remarkably, the p185^Bcr-Abl^-transformed cells with Abi1 deficiency lost their ability to develop leukemia in syngeneic mice. Even though these cells developed drug tolerance in vitro after prolonged selection with imatinib as their parental cells, the imatinib-tolerant cells remain incapable of leukemogenesis in vivo.

**Conclusions:**

Together, this study highlights an essential role of Abi1 in Bcr-Abl-induced leukemogenesis and provides a model system for dissecting the Abi1 signaling in Bcr-Abl-positive leukemia.

## Background

The *Bcr-Abl* oncogene is generated by a reciprocal t(9;22)(q34;q11) chromosome translocation known as *Philadelphia chromosome* (*Ph*), which fuses varying amounts of the *breakpoint cluster region* (*Bcr*) gene on chromosome 22 with sequences upstream of the second exon of *cellular Abl* (*cAbl*) gene on chromosome 9. Depending on the amount of *Bcr* sequences fused, three different Bcr-Abl fusion proteins may be produced with molecular masses of 185 kilodalton (Kd) (p185^Bcr-Abl^), 210 Kd (p210^Bcr-Abl^), and 230 Kd (p230^Bcr-Abl^) [1–3]. p210^Bcr-Abl^ expression is a causative event in over 95% of human chronic myelogenous leukemia (CML) cases, while p185^Bcr-Abl^ is found in 60–80% of *Ph*-positive B cell acute lymphocytic leukemia (*Ph*^*+*^ B-ALL) cases [3–5]. Development of the Abl tyrosine kinase inhibitor (TKI) imatinib and other second-generation TKIs, dasatinib and nilotinib, has revolutionized the treatment of *Ph*^*+*^ leukemia, with remarkable rates of sustained complete cytogenetic remission and disease-free survival for CML patients at the chronic phase [6]. However, relapse is often observed in the patients with *Ph*^*+*^ B-ALL or advanced CML due to the persistence of leukemic progenitor cells and accumulation of additional mutations that result in drug resistance [6–8]. A major challenge in the treatment of *Ph*^*+*^ leukemia has been in developing novel therapies for patients who are resistant to TKI-based therapy.

The hematopoietic stem/progenitor cells isolated from *Ph*^*+*^ leukemia patients exhibit multiple abnormalities of cytoskeletal function such as increased motility, altered adhesion, and decreased response to stromal cell-derived factor 1α (SDF-1α) [9–11]. These abnormalities may play a critical role in the progression of leukemia, since altered adhesion and mobility may contribute to premature release of leukemic stem/progenitor cells from bone marrow and accumulation and infiltration of these cells in peripheral hematopoietic tissues such as blood, spleen, and liver. Abnormal actin remodeling may also contribute to the deregulation of leukemic progenitor cell proliferation and survival [11]. Bcr-Abl oncoproteins exert their oncogenic potential in cooperation with additional cytoplasmic and nuclear effectors such as those involved in the regulation of mitogenic and apoptotic pathways [1, 5]. They are also capable of binding to cytoskeleton proteins and other proteins involved in the regulation of cell adhesion and migration [1, 5, 12]. Among these proteins is the Abl interactor 1 (Abi1) [13], a key regulator of Rac-dependent actin polymerization [14, 15]. Abi1 is present in cells as a complex with *WA*SP-family verprolin-homologous (WAVE) proteins, Nck-associated protein (Nap), specifically Rac-associated (Sra) protein, and hematopoietic stem progenitor cell 300 (Hspc 300) [14, 16–18]. The macromolecular complex, named WAVE regulatory complex (WRC), regulates initiation of actin polymerization in response to signal transduction from membrane receptors to small GTP-binding proteins and PI3 kinase (PI3K) [19–21]. In addition to the interactions with Abl, WAVE and Nap, Abi proteins were also found to interact with a variety of other signaling molecules that are involved in the control of cell proliferation, apoptosis, cytoskeletal functions, receptor signaling, endocytosis, and trafficking [19, 21–29]. Despite the importance of Abi1 in intracellular signaling, its role in cancer and leukemia development remains unclear. Previously, we have shown that the knockdown of Abi1 expression by sequence-specific small hairpin RNA (shRNA) inhibited p185^Bcr-Abl^-stimulated cell adhesion and migration in vitro and impaired p185^Bcr-Abl^-induced leukemogenesis in vivo [30, 31]. In these studies, however, the leukemogenesis was delayed but not eliminated, possibly due to incomplete Abi1 depletion [30]. In addition, studies by Chorzalska et al. suggest that the low expression of Abi1 may associate with drug resistance of Bcr-Abl-positive leukemic cells, whereas Juskevicius et al. reported that relapsing diffuse large B cell lymphoma (DLBCL) more commonly displayed gains of a cluster of genes including Abi1 [32, 33]. More recently, Chorzalska et al. reported that bone marrow-specific knockout of Abi1 induces myeloproliferative neoplasm [34]. Studies in other cancer cells involving the role of Abi1 in cancer development in vitro and in vivo are also contradictory. While the studies of breast cancer and colorectal carcinoma cells support a role of Abi1 in breast cancer and colorectal cancer development in vitro and in vivo [35–37], other studies suggest that Abi1 may function as a tumor suppressor in prostate cancer and gastric carcinoma development [38–40]. To determine the role of Abi1 in p185^Bcr-Abl^-positive leukemia development, we set to completely deplete its expression in p185^Bcr-Abl^-positive leukemic cells using CRISPR/Cas9-mediated gene editing. Here, we report that Abi1 is involved in regulation of the Bcr-Abl signaling to downstream pathways including mitogen-activated protein kinases (MAPK) and PI3K-Akt pathways. The complete depletion of Abi1 not only inhibits Bcr-Abl-induced abnormal actin polymerization, cell proliferation, and cell migration in vitro, but also inhibits leukemogenesis in vivo. Moreover, the inhibition of Bcr-Abl-induced leukemia by Abi1 deficiency is independent of the sensitivity of these cells to imatinib, as the imatinib-tolerant p185^Bcr-Abl^ cells also require Abi1 for development of leukemia in vivo.

## Materials and methods

### Cell lines and reagents

Ba/F3 cells were grown in RPMI containing 10% fetal bovine serum (FBS) and 15% WEHI3-conditioned medium as a source of IL3. The Ba/F3 cell lines expressing p185^Bcr-Abl^ with or without Abi1 deficiency were cultured in RPMI containing 10% FBS. The preparation of rabbit polyclonal antibodies against Abi1 and Abi2 has been described previously [41, 42]. The antibodies against Abl were purchased from Santa Cruz Biotechnology, Inc. (Santa Cruz, CA) and the rabbit monoclonal antibodies for pan- and phospho-Akt (Ser 473), p38 MAPK, phospho-p38 (Thr180/Tyr182), p42/44 ERK, and phospho-p42/44 ERK (Thr202/Tyr204) were obtained from the Cell Signaling Technology, Inc. (Danvers, MA). The monoclonal anti-β-actin antibody and the protease inhibitor cocktail were purchased from Sigma (St. Louis, MO).

### CRISPR/CAS9-mediated gene editing

**Fig. 1:**
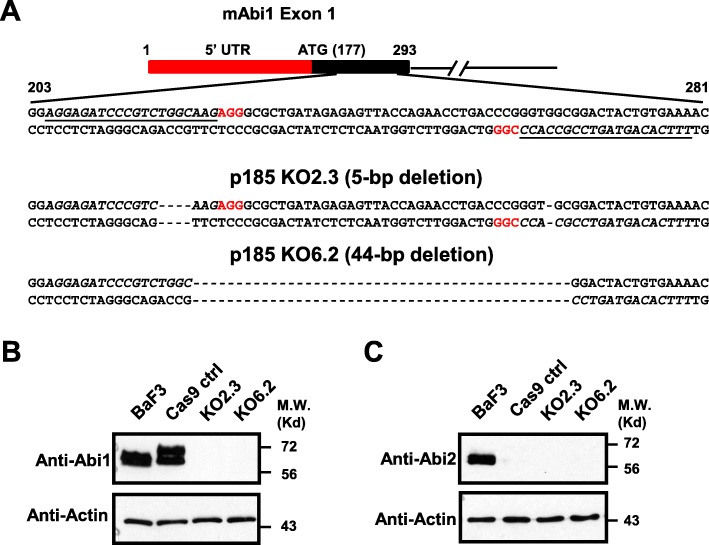
Generation of Abi1 deficient cell lines in p185^Bcr-Abl^-transformed BaF3 cells. **a**. Sequencing analysis of indel mutations in p185^Bcr-Abl^ Abi1 knockout cells. Sequences targeted by gRNA A and B are underlined and the PAM sequences are in red. **b**. Abi1 expression in Ba/F3, p185 control (Cas9 ctrl), and two independent p185^Bcr-Abl^ Abi1 knockout cell lines, KO2.3 and KO6.2. **c**. Abi2 expression in Ba/F3, Cas9 ctrl, KO2.3 and KO6.2 cells. Total lysates from 5 × 10^5^ cells of each cell line, as indicated, were subjected to Western blot analysis with indicated antibodies

To generate Abi1 deficient p185^Bcr-Abl^ cells, CRISPR/Cas9-mediated gene editing was performed. Both strands of oligo DNAs encoding for two gRNAs that specifically target Abi1 exon 1 sequences (gRNA A: 5′AGGAGATCCCGTCTGGCAAG3′ and gRNA B: 5′TTTCACAGTAGTCCGCCACC3′, Fig. 1) were designed using an online CRISPR design tool [43]. The two pairs of oligos were synthesized, annealed, and cloned into plasmid pSpCas9 (BB)-2A-Puro, a gift from Feng Zhang (Addgene plasmid no.48139; http://n2t.net/addgene:48139; RRID:Addgene_48139), respectively, at the BbsI site [43]. The resultant plasmids, pSpCas9 Abi1 KOA and pSpCas9 KOB, were amplified and transfected into p185^Bcr-Abl^-transformed Ba/F3 cells by electroporation. The transfected cells were serially diluted in 24-well plate in RPMI 1640 containing 10% fetal bovine serum (FBS) and 15% WEHI3-conditioned medium as a source of IL3. The stably transfected cell lines were selected by 2 μg/ml puromycin. The stably transfected clonal lines with complete depletion of Abi1 expression were initially identified by western blot analysis.

### Indel mutations analysis of Abi1 knockout cell lines

To analyze indel mutations in p185^Bcr-Abl^ Abi1 knockout cells, the genomic DNAs from the knockout clonal lines were purified using the Wizard Genomic DNA Purification kit (Promega, Madison, WI). Polymerization chain reaction (PCR) was then performed to amplify *ABI1* exon 1 using the genomic DNA as template and the following oligos as primers: forward 5′ GAGAGTAAGGAGGAAGAGGAGG 3′ and reverse 5′ GACCTCAGCCAGGGCAGGTGG 3′. The amplified DNA was digested by restriction enzyme and cloned to plasmid pBSK at the Sac I site. The resultant plasmids were sequenced to identify indel (Fig. 1).

### Biochemical assay

Western blot analyses were performed as previously described [44]. Briefly, control Ba/F3 cells and Ba/F3 cells expressing p185^Bcr-Abl^ with or without *ABI1* deficiency were lysed in lysis buffer (20 mM Hepes, pH 7.2; 150 mM NaCl, 1% Triton X-100, and 10% glycerol) and total cell lysates were separated on SDS-PAGE, transferred to nitrocellulose, and immunoblotted with appropriate antibodies. We used the ImageJ software program to quantify the levels of phosphorylated MAP kinases and Akt in three independent western blot assays.

### In vivo leukemogenesis studies

A suspension of 1X10^6^ Ba/F3 cells expressing p185^Bcr-Abl^ with or without *ABI1* deficiency was injected into 6–8 weeks old female BALB/c mice through the tail vein. Because Ba/F3 cells are considered syngeneic to BALB/c mouse, no irradiation was given to the recipient mice. The mice were followed for disease development, as judged by symptoms such as abnormal gait and labored breathing. Moribund animals were sacrificed by CO_2_ asphyxiation and were examined for tumors or other visible abnormalities. Collection of spleens and livers was performed immediately after sacrifice and the tissues were fixed. Tissue sections were prepared and haemotoxylin and eosin (H&E) stain of the sections was performed by Texas Veterinary Medical Diagnostic Laboratories. All protocols used were approved by Institutional Animal Review Committee at the Texas Tech University Health Sciences Center.

### Cell migration assay

The cell migration assay was performed as described previously [45]. Ba/F3 cells expressing p185^Bcr-Abl^ with or without *ABI1* deficiency were resuspended in RPMI 1640 medium at a concentration of 1 × 10^6^ cells/ml. A suspension of 0.1 ml cells was then added into the inserts of Transwell plates (8-μm pores, Corning Costar Corp., Cambridge, MA) and cells were allowed to migrate to the bottom chamber containing 0.6 ml RPMI 1640 with or without 50 ng/ml of SDF-1α at 37 °C in a 5% CO_2_ incubator for 12 h.

### Fluorescence microscopy and flow cytometry analysis

Cultured Ba/F3 cell lines expressing p185^Bcr-Abl^ with or without *ABI1* deficiency were fixed in 4% paraformaldehyde (PFA) in PBS for 10 min, permeabilized in 0.2% Triton X-100/PBS for 5 min, and stained with 50 μg/ml TRITC-conjugated phalloidin (Sigma, St. Louis, MO) in PBS. After washing cells extensively with PBS and briefly staining them with DAPI (Sigma, St. Louis, MO) to visualize nuclei, 5–10 × 10^3^ cells were loaded per slide by cytospin and mounted with Vectashield mounting medium (Vector, Burlingame, CA). Images were captured and analyzed using Olympus IX81 microscope with associated Image software.

### Statistical analysis

Descriptive statistics were generated for all quantitative data with presentation of means ± SDs. Significance of comparisons between experimental groups was tested using the Student’s *t* test.

## Results

### CRISPR/Cas9-mediated Abi1 gene editing in p185^Bcr-Abl^-transformed Ba/F3 cells

To determine the role of Abi1 in Bcr-Abl-induced cellular transformation and leukemogenesis, we used CRISPR/Cas9-mediated gene editing to deplete Abi1 gene expression in p185^Bcr-Abl^-transformed Ba/F3 cells (hereinafter referred to p185^Bcr-Abl^ cells). A mix of two plasmid DNAs, each containing a Cas9 gene and a gene encoding for a gRNA (gRNA A or B, Fig. 1a) that targets different regions in the mouse *ABI1* exon 1, was introduced into p185^Bcr-Abl^ cells. As a control, a plasmid expressing Cas9 only was also introduced into the p185^Bcr-Abl^ cells (hereinafter referred to p185 control cells). Insertion and deletion (Indel) mutation analysis identified two independent clonal lines, p185 KO2.3, which has a 5-base pair (bp) deletion, and p185KO6.2, which has a 44-bp deletion in the *Abi1* exon 1 (Fig. 1a). These deletions cause a reading frame shift and premature stop of Abi1 protein translation. Consistently, western blot analysis shows that the Abi1 expression is completely depleted in p185 KO2.3 and p185 KO6.2 cells as compared to p185 control cells (Fig. 1b).

Previously, we have shown that the expression of Bcr-Abl in hematopoietic cells induces the degradation of Abi2 through an ubiquitin-dependent proteolysis pathway [41]. To determine if Abi1 knockout affects Bcr-Abl-induced Abi2 degradation, we examined the protein level of Abi2 in p185 KO2.3 and KO6.2 cells. Consistent with the previous report [41], the expression of Abi2 is lost in p185 control cells as compared to parental Ba/F3 cells (Fig. 1c). Similarly, no Abi2 was detected in p185 KO2.3 and p185 KO6.2 cells (Fig. 1c), suggesting that Bcr-Abl-induced downregulation of Abi2 is not affected by Abi1 depletion.

### Knockout of Abi1 inhibited cell proliferation, SDF-induced chemotaxis, and invadopodia formation in p185^Bcr-Abl^-transformed Ba/F3 cells

**Fig. 2:**
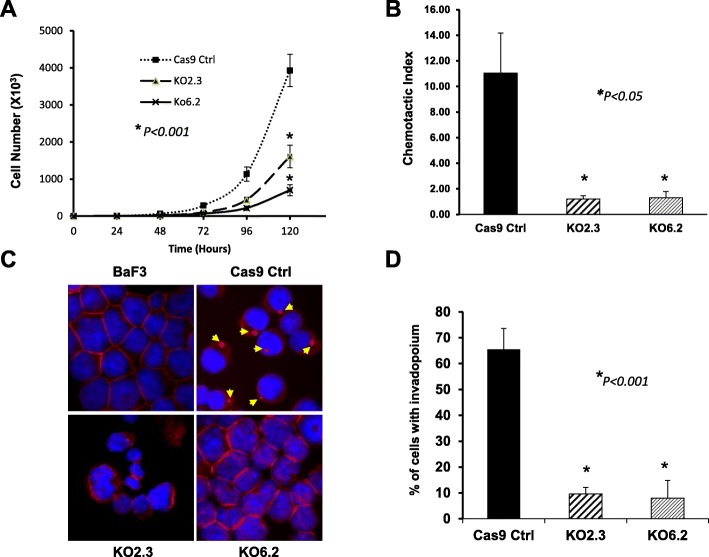
Effects of Abi1 deficiency on IL3-independent cell growth, SDF-induced chemotaxis, and F-actin remodeling of the p185^Bcr-Abl^-transformed Ba/F3 cells. **a**. IL3-independent growth of p185 Cas9 control cells (Cas9 Ctrl) and two p185 Abi1 knockout cell lines (KO2.3 and KO6.2). *P < 0.001 as compared to Cas9 Ctrl cells. **b**. Effects of Abi1 deficiency on SDF1α-induced chemotaxis. The p185 Cas9 control cells (Cas9 Ctrl) and two independent lines of p185 Abi1 knockout cells (KO2.3 and KO6.2) were tested in Transwell plate (1.0 × 10^5^ /insert) for SDF1α (50 ng/ml) stimulated migration. The vertical axis shows the chemotactic index expressed as the average ratio +/- S.D. of migrated cells in the presence of SDF1α to those in the absence of SDF1α. The data was calculated from triplicate wells from a representative assay of three independent experiments. *P < 0.05 as compared to Cas9 Ctrl cells. **c** and **d**. Abi1 is required for Bcr-Abl-induced abnormal F-actin remodeling. Ba/F3, Cas9 Ctrl, KO2.3, and KO6.2 cells, as indicated, were fixed and stained with TRITC-conjugated phalloidin for F-actin (red) and DAPI for nucleus (blue). The cells with F-actin rich invadopodium structures were visualized by fluorescence microscopy, as shown by arrowheads (C, Cas9 Ctrl panel) and were counted (D, expressed as average percentage +/- S.D. of three randomly picked areas). *P < 0.001 as compared to Cas9 Ctrl cells

Transformation of Ba/F3 cells by p185^Bcr-Abl^ resulted in interleukin 3 (IL3)-independent growth. Knockout of Abi1 in p185^Bcr-Abl^ cells did not abolish IL3-independent cell growth (Fig. 2a). However, Abi1 deficiency resulted in a slower cell growth of p185 KO2.3 and p185 KO6.2 cells (2.4-fold and 5.6-fold reduction, respectively) in IL3-free medium as compared to that of the p185 control cells (Fig. 2a).

To determine if the Abi1 depletion affects cell migration, we examined the SDF-1α-induced chemotaxis of the p185 KO2.3 and p185 KO6.2 cells using Boyden chamber transwell migration assay and compare it to that of p185 control cells. As shown in Fig. 2 b, addition of 50 ng/ml of SDF-1α in the bottom chamber stimulated p185 control cell migration by 11-fold as compared to that without SDF-1α. In contrast, 50 ng/ml SDF-1α failed to induce the p185 KO2.3 and p185 KO6.2 cells to migrate to the bottom chamber (Fig. 2b). Thus, our data suggests that the depletion of Abi1 in these cells inhibits the SDF-1α-induced chemotaxis.

We and others have previously shown that the expression of p185^Bcr-Abl^ in Ba/F3 cells induced a profound actin cytoskeleton remodeling and invadopodia formation [44, 46]. Specifically, an invadopodia structure characterized by intensively staining with phalloidin, indicative of filament actin (F-actin) aggregates, was observed in 66% of p185 control cells, but not in Ba/F3 cells (Fig. 2c). Depletion of Abi1 resulted in a 7- and 8-fold reduction, respectively, in this invadopodium formation in the p185 KO2.3 and p185 KO6.2 cells (Fig. 2c, d).

### Abi1 deficiency impaired the Bcr-Abl signaling to downstream pathways

**Fig. 3:**
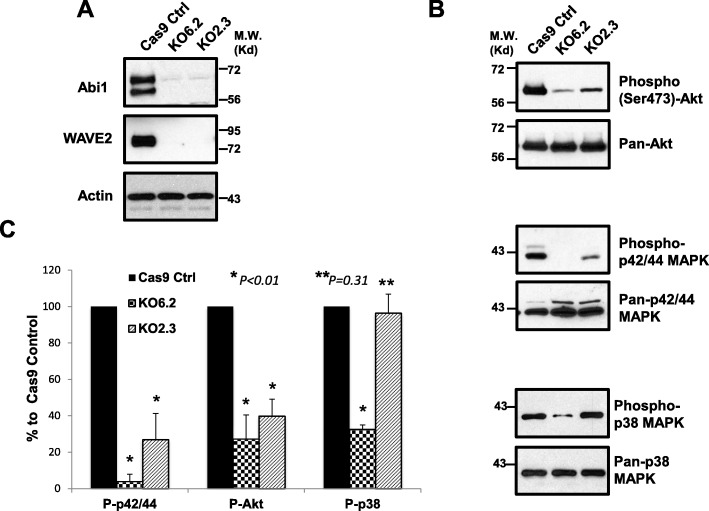
Abi1 deficiency in the p185^Bcr-Abl^-transformed Ba/F3 cells reduced WAVE2 expression and the Bcr-Abl signaling to MAPK and PI3 kinases. **a**. WAVE2 expression in p185^Bcr-Abl^ Cas9 control cells (Cas9 Ctrl) and two independent p185 Abi1 knockout cell lines (KO2.3 and KO6.2), as indicated. **b**. Effects of Abi1 deficiency on the p185^Bcr-Abl^ signaling to MAPK and PI3 kinases. Upper panel: Decreased Akt serine 473 phosphorylation in Abi1 deficient p185^Bcr-Abl^ cells. Middle panel: Abi1 deficiency inhibited p185^Bcr-Abl^-induced p42/44 ERK phosphorylation at threonine 202 and tyrosine 204. Bottom panel: Effects of Abi1 deficiency on p185^Bcr-Abl^-induced p38 MAPK phosphorylation at threonine 180 and tyrosine 182. The western blot shown is a representative of three independent experiments. **c**. Quantitative analysis of three independent western blots using ImageJ program. After normalized to their total protein, levels of the phosphorylated-p42/44 ERK (P-p42/44), Akt (P-Akt), and p38 MAPK (P-p38) in Cas9 Ctrl, KO6.2, and KO2.3 cells are expressed in the vertical axis as the average percentage +/- SD of that in Cas9 Ctrl cells. *P < 0.01 and **P = 0.31 as compared to Cas9 control cells

Abi1 is a component of WRC that regulates WAVE actin nucleation promoting activity and links WAVE to the Abl tyrosine kinases. To determine the effect of Abi1 depletion on the WRC signaling, we examined the protein level of WAVE2 in p185^Bcr-Abl^ Abi1 knockout cells. In line with the results reported by other investigators [15, 16, 27], the knockout of Abi1 in p185^Bcr-Abl^ cells resulted in a marked reduction of WAVE2 protein level (Fig. 3a).

Next, we tested if the Abi1 depletion affects the Bcr-Abl signaling to other downstream pathways. The mitogen-activated protein kinase (MAPK) signaling pathways and phosphatidylinositol 3-kinase (PI3K)-Akt pathway have been shown previously to be activated by Bcr-Abl and their activation plays a key role in Bcr-Abl-induced leukemogenesis [1, 5]. To determine if the Abi1 deficiency affects the Bcr-Abl signaling to MAPK pathways, we examined the activation of p38 MAPK and p42/44 extracellular signal–regulated kinases (p42/44 ERK) in p185 Abi1 knockout cells using antibodies that recognize phosphorylated and activated p38 MAPK and p42/44 ERK. As shown in Fig. 3 b and c, Abi1 depletion in p185 KO6.2 and p185 KO2.3 cells inhibited Bcr-Abl-induced p42/44 ERK activation by 96% and 73%, respectively, as compared to that in p185 Cas9 control cells. The Abi1 deficiency also decreased p38 MAPK activation by 67% in p185 KO6.2 cells as compared to that in p185 control cells (Fig. 3b, c). This decrease in p38 MAPK activation, however, was not observed in p185 KO2.3 cells (Fig. 3b, c). Because the previous studies have shown an association between Abi1 and PI3K [20, 47], we also examined the PI3K/Akt pathway using anti-phosphorylated Akt antibodies that recognize activated Akt. We found that the depletion of Abi1 in p185 KO6.2 and p185 KO2.3 cells reduced the Bcr-Abl-induced Akt activation by 73% and 60%, respectively, as compared to that in p185 Cas9 control cells (Fig. 3d). Taken together, our data supports a role of Abi1 in regulating the Bcr-Abl signaling to ERK and PI3K/Akt pathways.

### Abi1 is essential for Bcr-Abl-induced leukemogenesis in vivo

**Fig. 4:**
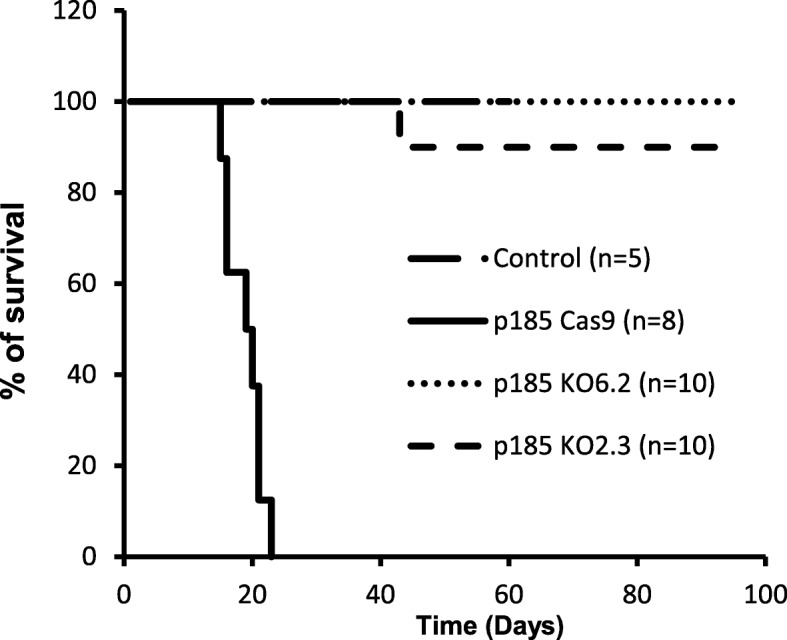
Abi1 deficiency abrogates the p185^Bcr-Abl^-induced leukemogenesis in vivo. Survival of the syngeneic Balb/C mice injected with saline as a control (control) or 1X10^6^ of p185^Bcr-Abl^ Cas9 (p185 Cas9), p185^Bcr-Abl^ KO2.3 (p185 KO2.3), and p185^Bcr-Abl^ KO6.2 (p185 KO6.2) cells, as indicated. Survival of the mice were monitored and represented as the percentage of survival

**Fig. 5:**
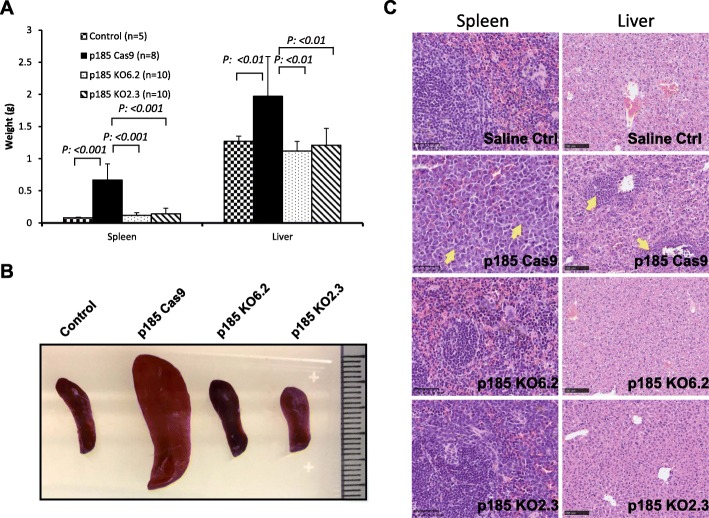
Pathology analysis of the syngeneic mice injected with saline, p185^Bcr-Abl^ Cas9, p185^Bcr-Abl^ KO2.3, and p185^Bcr-Abl^ KO6.2 cells. **a**. Liver and spleen weights of mice injected with saline (control), p185^Bcr-Abl^ Cas9 (p185 Cas9), p185^Bcr-Abl^ KO2.3 (p185 KO2.3), and p185^Bcr-Abl^ KO6.2 (p185 KO6.2) cells. **b**. Spleens from the mice received saline as control or p185 Cas9, p185 KO2.3, and p185 KO6.2 cells, as indicated. **c**. Histology of livers and spleens from the mice that received saline as control or p185 Cas9, p185 KO2.3, and p185 KO6.2 cells, as indicated. Livers and spleens were collected from moribund mice received p185 Cas9 control cells and the age-matched mice received p185 KO2.3, p185 KO6.2 cells, or saline as control. Collected tissues were fixed in 10% formalin for 24 h and then paraffin-embedded. The sections from embedded tissues were stained with hematoxylin and eosin. Arrows indicate the massively expanded p185 Cas9 control cells in spleen and liver that are morphologically distinguishable from normal tissue cells

Complete knockout of Abi1 expression in p185^Bcr-Abl^ cells allowed us to test if Abi1 is required for Bcr-Abl-induced leukemogenesis in vivo. To this end, we injected p185^Bcr-Abl^ control, two independent lines of p185 Abi1 knockout cells p185 KO2.3 and p185 KO6.2, or saline as control into syngeneic Balb/C mice through tail vein. All recipient mice were then followed for the development of leukemia. As reported previously, while the control mice injected with saline were healthy with no sign of disease for up to 6 months, the mice injected with p185^Bcr-Abl^ control cells developed leukemia in 2 to 3 weeks. These mice either died or became moribund with a mean survival of 18.9 days (Table 1 and Fig. 4). In contrast, all the mice injected with p185 KO 6.2 cells and 70% of the mice injected with p185 KO 2.3 cells were healthy with no signs of disease for up to 6 months (Table 1 and Fig. 4). Only one out of ten mice injected with p185 KO 2.3 cells developed leukemia approximately 4-month post-transplantation and two others developed solid tumors around chest (the mouse p185 KO2.3 A2, Table 1) and gastrointestinal tissues (the mouse p185 KO2.3 A1, Table 1), respectively. Gross pathology analysis revealed that all the mice injected with p185 control cells developed splenomegaly and hepatomegaly (Table 1 and Fig. 5a, b), whereas no apparent splenomegaly nor hepatomegaly was observed in all mice injected with p185 KO 6.2 cells and 90% of the mice injected with p185 KO 2.3 cells (Table 1 and Fig. 5a, b). Histopathology analysis showed that the destruction of normal cytoarchitecture in the spleen and liver due to the massive accumulation of p185^Bcr-Abl^-positive leukemic cells which are morphologically distinguishable from normal cells, was observed in the mice injected with p185 control cells (Fig. 5c). In contrast, no apparent abnormality of the splenic and hepatic cytoarchitecture was observed in the mice injected with p185 Abi1 knockout cells (Fig. 5c).

**Table 1:** Summary of the disease development in mice injected with p185 control cells and the p185 KO2.3 cells

Mouse	Latency ^*a*^(days)	Spleen weight (g)	Liver weight (g)
**Saline Ctrl**			
A1	60^*b*^	0.07	1.14
A2	125^*b*^	0.08	1.30
A3	125^*b*^	0.08	1.36
A4	181^*b*^	0.10	1.30
A5	181^*b*^	0.09	1.27
**p185 Cas9**			
A1	19^*c*^	0.53	2.99
A2	20^*c*^	0.74	2.77
A3	21^*c*^	0.74	1.86
A4	23^*c*^	1.05	1.49
A5	21^*c*^	0.98	1.48
B1	15^*c*^	0.44	1.88
B2	16^*c*^	0.47	2.01
B3	16^*c*^	0.39	1.28
**p185 KO2.3**			
A1	43^*d*,*e*^	0.09	1.1
A2	157^*c*,*e*^	0.10	0.77
A3	181^*b*^	0.11	1.33
A4	181^*b*^	0.12	1.41
A5	181^*b*^	0.14	1.73
B1	102^*b*^	0.08	1.08
B2	116^*c*^	0.40	0.94
B3	184^*b*^	0.10	1.22
B4	184^*b*^	0.11	1.26
B5	184^*b*^	0.11	1.27

^*a*^Latency is defined as the time post-injection that mice died or become moribund

^*b*^The day euthanized without any sign of disease

^*c*^The mice found moribund at the day of pathology analysis

^*d*^The mouse found dead at the day of pathology analysis

^*e*^Tumors were found around gastrointestinal tissue (A1) and in chest (A2) of mice

### Abi1 is required for leukemogenic activity of imatinib-tolerant p185^Bcr-Abl^ cells

**Fig. 6:**
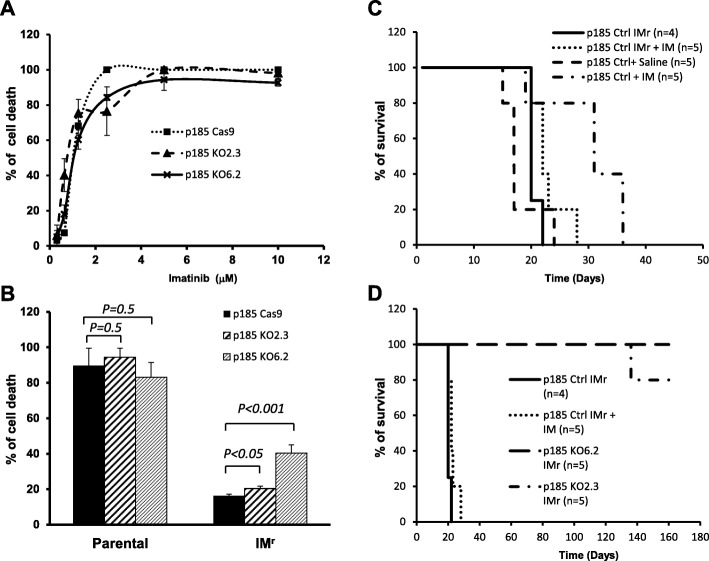
Effect of imatinib treatment on IL3-independent growth and leukemogenesis of Abi1-deficient p185^Bcr-Abl^ cells. **a**. Imatinib induces dose-dependent cell death in control p185^Bcr-Abl^ cells (p185 Cas9) and Abi1-deficient p185^Bcr-Abl^ cells (p185 KO2.3 and p185 KO6.2). The p185 Cas9, p185 KO2.3, and p185 KO6.2 cells grown in IL3-free growth medium were treated with imatinib at 0.31, 0.63, 1.25, 2.5, 5, and 10 μM, as indicated, for 48 h, cell viability was determined by trypan blue exclusion assay and represented as average +/- SD of triplicate wells. **b**. Imatinib-induced cell death in parental and imatinib-tolerant (IM^r^) p185 Cas9, p185 KO2.3, and p185 KO6.2 cells. The p185 Cas9, p185 KO2.3, and p185 KO6.2 cells are selected without (parental) or with 1.25 μM imatinib (IM^r^) for six weeks. The cells were then treated with 1.25 μM imatinib for 72 h in IL3-free growth medium. The cell viability was determined by trypan blue exclusion assay and represented as mean +/- SD of triplicate wells. **c**. Effect of imatinib treatment on the survival of syngeneic mice injected with the control p185^Bcr-Abl^ (p185 Ctrl) and imatinib-tolerant p185^Bcr-Abl^ (p185 IMr) cells. The Balb/C mice were injected through tail vein with 1X10^6^ p185 Ctrl or p185 IMr cells, as indicated. Ten days post-injection the mice were administered intraperitoneally once a day with either saline as control or imatinib (IM, 100 mg/Kg body weight), as indicated, for 5 consecutive days. Survival of the mice were monitored and expressed as the percentage of survival. **d**. The Abi1 deficient p185^Bcr-Abl^ cells tolerant to imatinib failed to develop leukemia in syngeneic mice. The Balb/C mice were injected through tail vein with 1X10^6^ imatinib-tolerant p185^Bcr-Abl^ control (p185 Ctrl IMr) cells as well as imatinib-tolerant p185 KO2.3 and p185 KO6.2 cells (p185 KO2.3 IMr and p185 KO6.2 IMr). Ten days post-injection the mice injected with p185 Ctrl IMr cells were administered intraperitoneally once a day with imatinib (IM, 100 mg/Kg body weight) for 5 consecutive days. Survival of the mice were monitored and represented as the percentage of survival

While the therapies using Bcr-Abl tyrosine kinase inhibitors achieve deep remissions and long-term survival for CML patients, overall survival of the patients with p185^Bcr-Abl^-positive ALL is still low and drug resistance is more frequently observed [6–8]. Treatment of p185^Bcr-Abl^-transformed Ba/F3 cells with imatinib for 48 h resulted in over 99% and 90% cell death in p185 control cells and in p185 Abi1 knockout cells, respectively (Fig. 6a). However, some cells survived and grew up after prolonged treatment. To determine if Abi1 deficiency in p185^Bcr-Abl^ cells affects their sensitivity to imatinib, we selected the p185 Cas9 and p185 Abi1 knockout cells that are resistant to imatinib by culturing these cell in 1.25 μm imatinib, a dosage that leads to 90 % cell death of p185 control cells within 72 h (Fig. 6b), for over 6 weeks. Under this selection, a small portion of p185 control and p185 Abi1 KO cells survived and eventually expanded. These cells, referred as imatinib resistant cells (IM^r^), were then examined for growth and survival at the presence of imatinib. As shown in Fig. 6 b, while imatinib treatment resulted in 90% cell death of parental p185 control cells in 72 h, only 16% cell death was observed in imatinib-resistant p185 control cells (p185 control IM^r^) under the same condition. Like p185 control cells, imatinib treatment led to 94% and 83% cell death of p185 Abi1 KO 2.3 and KO 6.2 cells in 72 h, respectively (Fig. 6b). The imatinib-resistant p185 Abi1 KO 2.3 and KO 6.2 cells, however, showed a higher sensitivity to imatinib than that of p185 control IM^r^ cells, as the imatinib treatment resulted in greater cell death (20.4% and 40.4%, respectively) in these cells compared to that observed in p185 control IM^r^ cells (Fig. 6b).

The imatinib tolerance of p185 control IM^r^ cells was also observed in vivo (Fig. 6c). The mice injected with parental p185 control cells or p185 control IM^r^ cells both developed leukemia and died around 3–4 weeks post-injection. A 5-day treatment of mice with imatinib improved the survival of the mice injected with p185 control cells to 5 weeks. However, the treatment failed to extend the survival of the mice injected with p185 control IM^r^ cells, as these mice died in 3–4 weeks (Fig. 6c). To determine if the imatinib-insensitive p185 KO2.3 IM^r^ cells and p185 KO 6.2 IM^r^ cells regain the leukemogenic activity in vivo, we injected these cells into mice and monitored leukemia development. In contrast to the mice injected with p185 control IM^r^ cells, which developed leukemia within 3–4 weeks regardless whether treated with or without imatinib (Fig. 6d), all mice injected with p185 KO6.2 IM^r^ and 4 out of 5 mice injected with p185 KO2.3 IM^r^ showed no sign of disease for over 5 months. One out of 5 mice injected with p185 KO2.3 IM^r^ developed leukemia with much prolonged latency (> 4 months). Taken together, our data suggests that Abi1 is also essential for leukemia development in imatinib-tolerant p185^Bcr-Abl^ cells.

## Discussion

To dissect how Abi1 functions in Bcr-Abl-induced leukemogenesis, we knocked it out in p185^Bcr-Abl^ Ba/F3 cells by CRISPR/Cas9-mediated gene editing. Two independent knockout cell lines were obtained. Complete depletion of Abi1 in these cells is confirmed by nucleotide insertion/deletion (indel) analysis as well as by protein expression analysis. In these cells, the expression of Abi2 is also dramatically downregulated due to Bcr-Abl-induced activation of proteolytic pathways, as we previously described [41]. Complete depletion of Abi1 and markedly reduced expression of Abi2 in these cells provide a simplified system for analysis of the role of the Abi pathway in Bcr-Abl-induced leukemogenesis.

We have previously knocked down the expression of Abi1 in p185^Bcr-Abl^ cells by short hairpin RNA (shRNA)-mediated gene silencing [30]. We have observed that reduced expression of Abi1 inhibited Bcr-Abl-stimulated invadopodia formation and cell migration in vitro. In line with these previous studies, we show here that complete Abi1 depletion abrogates Bcr-Abl-induced invadopodia formation and cell migration. Although the studies with shRNA-mediated gene silencing also suggested that Abi1 might be essential for p185^Bcr-Abl^-induced leukemogenesis, a definitive conclusion could not be made because of incomplete depletion of Abi1. Specifically, despite the prolonged latency, the mice receiving p185^Bcr-Abl^ cells in which Abi1 expression had been knocked down eventually developed leukemia and became moribund approximately 5-week post-injection [30]. The leukemia development in these mice is likely due to the selective expansion of those p185^Bcr-Abl^ cells in which Abi1 has not been knocked down, as suggested by the analyses of both the Abi1 expression in p185^Bcr-Abl^ cells recovered from leukemic mice and the competitive in vivo expansion assay [30]. This is further supported by the studies presented here. Remarkably, we found that complete depletion of Abi1 abolishes the leukemic potential of p185^Bcr-Abl^ cells and the mice implanted with these cells are leukemia free for over 6 months.

Abi1 is a component of WRC, a key regulator of actin dynamics in the leading edge of motile cells that plays a critical role in cell adhesion, migration, and invasion. In addition to its association with WRC, Abi1 also interacts with a variety of important signaling molecules downstream of receptor tyrosine kinases, including Abl, Cbl, Sos, Eps8, and the p85 regulatory subunit of PI3K [19–21, 25, 48]. It has been reported that, as a subunit of WRC, Abi1 interacts with diverse receptors and links them to the actin cytoskeleton [28, 29]. The ability to interact with the regulatory machinery of actin assembly as well as diverse signaling molecules and membrane receptors places Abi1 at a central position in the signaling network that integrates signals from membrane receptors to cytoskeletal functions. Consistent with this notion, knockout of Abi1 expression in mice leads to lethality in the early embryo stage [49, 50]. More recent studies by Chorzalska et al. show that bone marrow (BM)-specific loss of Abi1 results in abnormal hematopoietic cell development including anemia, premature exhaustion of BM hematopoietic stem cells, myeloproliferative neoplasm, and defects in B cell development [34]. Similar phenotypes were also observed in an earlier study by Park et al. in which they knocked out Hem1, another component of WRC, in mice [51]. They show that depletion of Hem1 resulted in degradation of Abi1 and WAVE2. Remarkably, Hem1-deficient mice also exhibit anemia, lymphopenia, neutrophilia, and defects of lymphoid B and T cell development. More recently, Shao et al. show that Hem1 and WRC are required for transition of fetal liver hematopoiesis to BM [52]. These studies are consistent with our findings that Abi1 is essential for the p185^Bcr-Abl^ signaling and leukemogenesis in a transformed pro-B cell line. Taken together, our studies and those of others highlight an important role of Abi1 in hematopoietic cell development, homeostasis, and leukemogenesis.

The complete depletion of Abi1 in p185^Bcr-Abl^ cells allows for loss of function analysis of Abi1 in the Bcr-Abl signaling and this has led to the findings that would be difficult to be uncovered by the analysis of reduced expression of Abi1. In previous studies, we found that, while partial depletion of Abi1 in p185^Bcr-Abl^ cells by shRNA-mediated gene silencing impaired these cells expansion in vivo, it did not affect Bcr-Abl-induced IL3-independent growth in vitro [30]. This is in contrast with present studies which show that the complete depletion of Abi1 not only abrogates leukemia development in vivo but also reduces IL3-independent growth in vitro. It is likely that the low expression of Abi1 in Abi1-knockdown cells may exert a growth disadvantage in an in vivo environment but it may not be sufficient to cause growth inhibition in vitro. Chorzalska et al. have reported that low expression of Abi1 in CD34+ cells from CML patients and K562 CML cell line is linked to drug resistance and is associated with elevated activation of ERK and Akt, the pathways that are activated by Bcr-Abl and are important for Bcr-Abl-induced cell growth and leukemia development [32]. In p185^Bcr-Abl^ Abi1 knockout cells examined in our studies, however, complete depletion of Abi1 decreased ERK and Akt activation. The reduced ERK and Akt activity in p185^Bcr-Abl^ Abi1 KO cells is consistent with the finding that these cells grow slower in vitro and fail to develop leukemia in vivo. To test whether the Abi1 depletion links to the drug resistance, we examined the effect of imatinib on growth and survival of p185^Bcr-Abl^ cells and p185^Bcr-Abl^ Abi1 knockout cells. Our data suggests that the Abi1 pathway is essential for p185^Bcr-Abl^-induced leukemia development regardless whether these cells develop imatinib resistance or not. Ba/F3 is a mouse pro-B cell line and the expression of p185^Bcr-Abl^ in Ba/F3 cells results in IL3-independent growth. The p185^Bcr-Abl^ transformation of Ba/F3 cells also induces abnormal actin cytoskeleton remodeling and Abi2 degradation. It is possible that the role of Abi1 in Bcr-Abl-induced leukemia development may vary among different hematopoietic cell lineages. In this regard, it is notable that although low expression of Abi1 is associated with increased activation of ERK and Akt pathways in hematopoietic cells from CML patients and the K562 CML cell line, such a converse correlation was not observed in Bcr-Abl-transformed Ba/F3 cells [32]. Moreover, while the BM-specific Abi1 deficiency leads to myeloproliferative neoplasm, it impairs B cell development in mice [34]. Further investigation is therefore needed to elucidate whether the Abi1 signaling functions differentially in different hematopoietic lineages.

## Conclusions

In summary, studies presented here reveal that Abi1 is required for the leukemogenic activity of a p185^Bcr-Abl^-transformed mouse pro-B cell line. Complete depletion of Abi1 leads to not only an inhibition of Bcr-Abl-induced actin cytoskeletal functions but also a decrease in IL3-independent growth. Decreased cell proliferation in Abi1-deficient p185^Bcr-Abl^ cells correlates with a reduced activity of MAPK and PI3K/Akt pathways. Importantly, we found that Abi1 is essential for the leukemogenic activity of p185^Bcr-Abl^-transformed cells regardless whether these cells developed imatinib resistance. Taken together, our data suggest that Abi1 may serve as a potential therapeutic target for p185^Bcr-Abl^-positive B-ALL.

## Data Availability

All data generated or analyzed during this study are included in this published article.

## Abbreviations

Abi1: Abl interactor 1
B-ALL: B cell acute lymphocytic leukemia
BM: Bone marrow
CML: Chronic myelogenous leukemia
DLBCL: Diffuse large B cell lymphoma
IL-3: Interleukin 3
IM: Imatinib
KO: Knockout
MAPK: Mitogen-activated protein kinase
*Ph*: Philadelphia chromosome
PI3K: Phosphoinositide 3-kinase
SDF: Stromal cell-derived factor
shRNA: Short hairpin RNA
TKI: Tyrosine kinase inhibitor
WRC: WAVE regulatory complex
